# Evaluation of antiproliferative and anti-inflammatory activities of methanol extract and its fractions from the Mediterranean sponge

**DOI:** 10.1186/1475-2867-12-18

**Published:** 2012-05-15

**Authors:** Afef Dellai, Monia Deghrigue, Audrey Laroche-Clary, Hedi Ben Masour, Nabil Chouchane, Jacques Robert, Abderrahman Bouraoui

**Affiliations:** 1 Laboratoire de Pharmacologie des Médicament Anticancéreux, Université Victor Segalen Bordeaux 2, Institut Bergonie, 229 cours de l’Argonne, Bordeaux Cedex, 33076, France; 2 Unité de Recherche des Substances Actives Marines (URSAM), Laboratoire de Pharmacologie, Faculté de Pharmacie, Avenue Avicenne, Monastir, 5000, Tunisie; 3 Laboratoire de biotechnologie et Valorisation de Bio Géo Ressources Institut Supérieur de Biotechnologie, ISBST BioTechPole Sidi Thabet Université Manouba, Ariana, 2020, Tunisie

**Keywords:** Spongia officinalis, Anti-inflammatory activity, Antiproliferative activity

## Abstract

**Background:**

Without doubt, natural products have been, and still are, the cornerstone of the health care armamentarium. Of all natural sources, the marine environment is clearly the last great frontier for pharmaceutical and medical research.

**Methods:**

This work progresses in the direction of identifying component(s) from the Mediterranean sponge, *Spongia officinalis* with pharmacological activities. In the present study we investigated the efficacy of methanol extract and its semi-purified fractions (F2, F3) from *Spongia officinalis* for their in vivo anti-inflammatory activity using the carrageenan-induced paw edema in rats and their in vitro antiproliferative effects by their potential cytotoxic activity using the MTT colorimetric method and clonogenic inhibition against three human cancer cell lines (A549, lung cell carcinoma, HCT15, colon cell carcinoma and MCF7, breast adenocarcinoma).

**Results:**

The fractions F2 and F3 showed interesting anti-inflammatory and antiproliferative activities in a dose dependent manner.

**Conclusions:**

The present study indicates that the methanolic extrac and its fractions from *Spongia officinalis* are a significant source of compounds with the antiproliferative and anti-inflammatory activities, and this may be useful for developing potential chemopreventive substances.

## Introduction

A variety of ingredients of traditional medicines and herbs are being widely investigated in several parts of the world to analyze their potential as therapeutic agents [1-3]. Since the few last decades, marine environment have been recognized to be a rich sources of bioactive metabolites with varied biological and pharmacological activities [4,5]. The most interesting phyla with respect to pharmacological active marine compounds include bacteria, fungi, algae, soft corals and gorgonians, sea hares and nudibranchs, bryozoans, tunicates and especially sponges [6]. Marine sponges have been considered as a gold mine during the past few decades with respect to the diversity of their secondary metabolites and continue to provide novel natural products with a remarkable chemical diversity. At present, there are a number of compounds from marine origin which are under investigation and/or are being developed as new pharmaceuticals on anticancer therapies [7,8]. A number of compounds possess antispasmodic, antimalarial, antifungal, antifouling, insecticidal, antiviral [7], antibacterial [9] and anti-inflammatory activities [10]. The objective of the present study was to evaluate the potency of methanol extract and its semi-purified fractions (F2, F3) from *Spongia officinalis* for inhibiting inflammation induced by carrageenan and for growth and clonogenic inhibiting of three human cancer cell lines A549, HCT15 and MCF7 with the aim of identifying novel molecules with interesting and potentially useful pharmacological activities.

## Materials and methods

### Sample collection and preparation of the methanol extract

The marine sponge, *Spongia officinalis* was collected from the Mediterranean Sea, in various areas of the coastal region of Monastir (Tunisia), in July 2010, at a depth between 2 and 5 meters. The collected samples were cleaned by rising with sea water and distilled water and transported in cool box to the laboratory where they are kept in a freezer (−20°C). Identification of specimen was carried out in the National Institute of Marine Sciences and Technologies, Salamboo, Tunisia.

The samples were defrosted, macerated in distilled water and then air dried at 30°C and finely powdered. 600 g of finely powdered sponge material were packed in small bags (5x10 cm) of Whatman filter paper No. 1 and all bags were sealed and soaked in a methanol bath three times, steeping for 48 h. The methanol extracts were combined and evaporated under vacuum at low temperature (<40°C) and then stored at −20°C until use.

### Purification of the methanol extract

In order to localize the active fraction, methanol extract of *Spongia officinalis* was purified, using C_18_ cartridges (Sep-pack, Supelco), by gradient elution with methanol–water mixture (0%, 50% and 80% methanol) to give 3 fractions (F1, F2 and F3). Methanol solvent was removed from fractions recuperated using rotating evaporator at 35°C and distilled water was then added to the residues and the aqueous phases were lyophilized. The powdered fractions were stored at −20°C until use.

Methanol extract, F2 and F3 fractions were diluted to the desired final concentration immediately prior manipulation.

### Animals

For the anti-inflammatory evaluation of the methanol extract and its semi-purified fractions (F2, F3), adult Wistar rats (150-180 g) of both sex, provided from Pasteur institute (Tunis, Tunisia) were used. All animals were fed a standard diet ad libitum and allowed free access to drinking water. Animals fasted overnight before any experiments. Housing conditions and in vivo experiments were approved according to the guidelines established by the European Union on Animal care (CEE Council 86/609) [11].

### Carrageenan induced rat paw edema

The anti-inflammatory activity of our extract and fractions on carrageenan-induced paw edema was determined according to Winter et al. [12]. The animals were divided into three groups consisting of 6 rats each. The control group received 2.5 ml/kg intraperitoneally (i.p.) of saline solution, the standard groups received Acetylsalicylate of Lysine (ASL) (300 mg/kg) (i.p.) and the test group received the methanol extract of *Spongia officinalis* (25, 50 and 100 mg/kg) and its semi-purified fractions (F2, F3) at 50 mg/kg (i.p.). 30 min after intraperitoneal administration of different substances, 0.05 ml of 1% carrageenan suspension was injected to all animals in the left hind paw. The paw volume up to the tibiotarsal articulation was measured using Plethysmometer (model 7150, Ugo Basile, Italy). The measures were determined at 0 h (V_0_) (before carrageenan injection) and 1, 3 and 5 h later (V_T_). The volume of paw swelling was determined for each rat and the difference between V_T_ and V_0_ was taken as the edema volume. The percentages of inhibition were calculated according to the following formula:

(1) $$\%inhibition={\left(V_{T}-V_{0}\right)}_{\mathrm{control}}-{\left(V_{T-}V_{0}\right)}_{treated\;group}/{\left(V_{T}-V_{0}\right)}_{\mathrm{control}}*100$$

### Cell culture

The human tumor cell lines A549 (lung cell carcinoma), HCT15 (colon cell carcinoma) and MCF7 (breast adenocarcinoma) were obtained from the American Type Culture Collection (ATCC, Manassas, VA). Cells were routinely grown with DMEM supplemented with 10% fetal calf serum and 1% penicillin/streptomycin, all obtained from Biochrom AG (Berlin, Germany). They were grown on Flasks (Nunc, Denmark) at 37°C in a humidified atmosphere containing 5% CO_2_. Cells were replicated every 4–5 days and the medium changed once in-between.

### Viability assay

The potential effects on cell viability were investigated according to previously reported conditions [13,14], using the MTT assay [3-(4,5-dimethylthiazol-2-yl)-2,5-diphenyl tetrazolium bromide, Sigma-Aldrich Chimie, Saint-Quentin-Fallavier, France] as an indicator of metabolically active cells [15].

Known number of A549, HCT15 or MCF7 cells (10^3^) were transferred into 96-well plates (Nunc, Denmark) in a volume of 200 μl of culture medium and incubated for 24 h before addition of test compounds. Cells were then exposed for 24 h at 37°C to known concentrations of the methanol extract or fractions to be tested. After drug exposure, the cells were washed with phosphate-buffered saline and then reincubated in fresh culture medium for a further 48 h, then the culture medium was removed and 200 μl of MTT reagent (diluted in culture medium, 0.5 mg/ml) was added. Following incubation for 4 h, the MTT/medium was removed and DMSO (200 μl) was added to dissolve the formazan crystals. Absorbance of the colored solution was measured on a microplate photometre (Bio-Tek Instruments) using a test wavelength of 570 nm and a reference wavelength of 630 nm. Results were evaluated by comparing the absorbance of the treated cells with the absorbance of wells containing cell treated by the solvent control. Conventionally, cell viability was estimated to be 100% in the solvent control. All experiments were performed at least twice in triplicate. The concentration of substance required for 50% growth inhibition (IC50) was estimated according to the method described by Dellai [5].

### Clonogenic inhibition assay

The clonogenic inhibition assay was performed as described previously by Nicolas et al. [16] with some modifications. Known number of A549, HCT15 or MCF7 cells (2.10^4^) were transferred into six-well plates (Becton Dickinson Labware, USA) in a volume of 2 ml of culture medium and incubated for 24 h before addition of test compounds. Cells were then exposed for 24 h at 37°C to known concentrations of the compound to be tested. After drug exposure, the cells were washed with phosphate-buffered saline and subsequently re-plated in appropriate dilution in triplicate to assess clonogenic ability. After incubation for 14 days, each plate was stained with crystal violet and colonies were counted with a “colony counter pen”. The surviving fraction was calculated as the ratio of the number of colonies formed after treatment to the product of the number of cells plated and the plating efficiency. The IC_50_ value is the concentration of the drug which is capable of bringing about 50% inhibition of colony formation.

### Statistical analysis

For all our experiments, a one-way ANOVA was used to analyze the differences between groups, followed by a Duncan’s test with a threshold of significance of p < 0.01 and p < 0.001 to detect specific differences, using a statistical software package (STATISTICA edition 99 Maisons-Alfor- France). We used this post hoc test or multiple comparison tests, to determine the significant differences between a single control group mean and the remaining treatment group means in an analysis of variance setting.

## Results

### Evaluation of anti-inflammatory activity

Results of the carrageenan induced rat paw edema are shown in Table 1. The i.p. administration of the methanol extract of *Spongia officinalis* (25, 50 and 100 mg/kg) produced a significant reduction of the edema throughout the entire period of observation in a dose related manner. Interestingly the highest reduction of the edema was at 3 h with respectively 42.5, 52.7 and 61.71%. After i.p. administration of the semi-purified fractions F2 and F3 obtained by fractionation of the methanol extract, significant activity was observed with fraction F3 at the dose of 50 mg/kg, at the third hour after carrageenan injection, with 72.85% reduction in paw volume, whereas at the same time, F2 inhibited edema by 58.05. Standard drugs, ASL (300 mg/kg), decreased paw edema by 62.3% at the third hour (Table 1). The present results indicate that methanol extract and its semi-purified fractions exhibit anti-inflammatory effects.

**Table 1:** **Anti-inflammatory effect of the intraperitoneal administration of methanol extract and its semi-purified fractions (F2-F3) of*Spongia officinalis*in Carrageenan-induced rat paw edema test**

**Treatment Dose (mg/kg)**		**Edema (10^-2^ml)**			**Edema inhibition (%)**		
		**1h**	**3h**	**5h**	**1h**	**3h**	**5h**
		1h	3h	5h	1h	3h	5h
Control	-	20.08±6.5	60.49±1.3	59.99±0.8	-	-	-
Methanol extract	25	17.46±5.1^ns^	34.78±5.3^**^	40.49±6.17^**^	13.04	42.5	32.5
	50	16.02±0.78^*^	28.61±1.29^**^	32.99±1.26^**^	20.17	52.7	45
	100	16.33±5.2^*^	23.16±1,14^**^	26.87±1.16^**^	18.67	61.71	55.20
Fraction F2	50	17.12±1.37^ns^	25.37±0.6^**^	29.12±4.17^**^	14.74	58.05	51.45
Fraction F3	50	13.5±2.9^**^	16.42±2.5^**^	21.7±3.55^**^	32.76	72.85	63.82
ASL (Reference drug)	300	13.71±3.27^**^	22.80±0.8^**^	22.83±4.12^**^	31.72	62.3	61.94

Values are expressed as mean±s.e.m. *P<0.01, **P<0.001, ns: not significant. n=6 animals.

### Evaluation of antiproliferative activity against tumor cell lines

**Figure 1:**
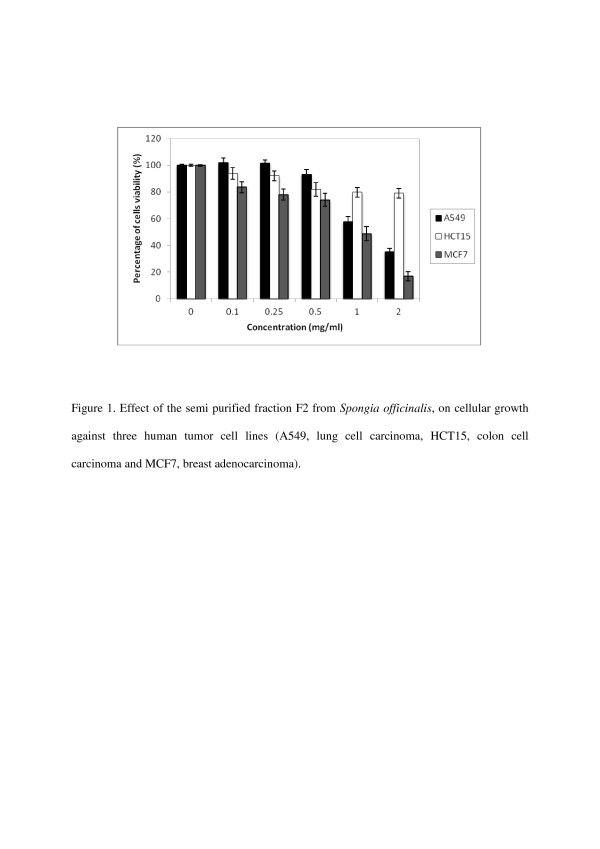
**Effect of the semi purified fraction F2 from*Spongia officinalis*, on cellular growth against three human tumor cell lines (A549, lung cell carcinoma, HCT15, colon cell carcinoma and MCF7, breast adenocarcinoma).**

**Figure 2:**
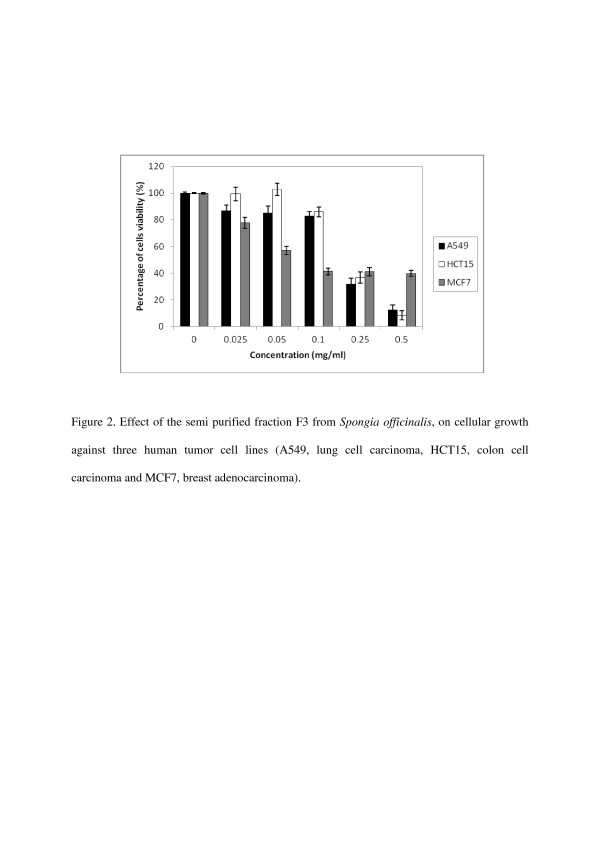
**Effect of the semi purified fraction F3 from*Spongia officinalis*, on cellular growth against three human tumor cell lines (A549, lung cell carcinoma, HCT15, colon cell carcinoma and MCF7, breast adenocarcinoma).**

**Figure 3:**
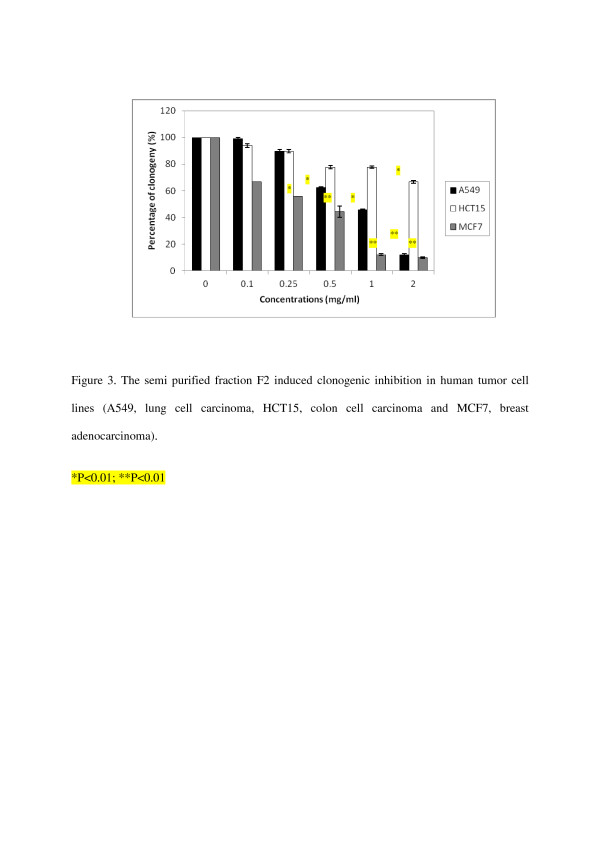
The semi purified fraction F2 induced clonogenic inhibition in human tumor cell lines (A549, lung cell carcinoma, HCT15, colon cell carcinoma and MCF7, breast adenocarcinoma).

**Figure 4:**
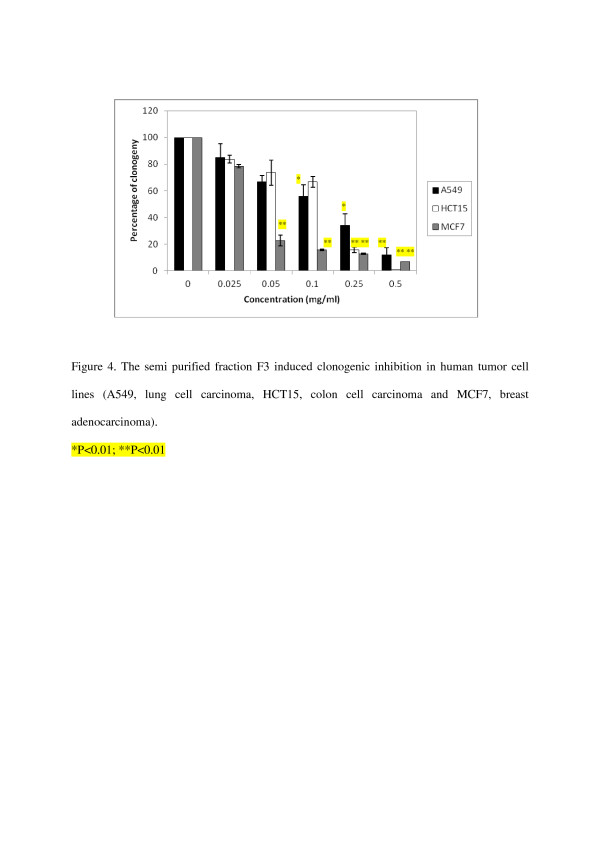
The semi purified fraction F3 induced clonogenic inhibition in human tumor cell lines (A549, lung cell carcinoma, HCT15, colon cell carcinoma and MCF7, breast adenocarcinoma).

In the first experiment, F2 was tested for its effect on inhibition of cell growth against three human tumor cell lines A549, HCT15 and MCF7 over a concentration range (100–2000 μg/ml) to determine their potency (IC_50_-50% inhibition of cell growth), results are shown in Table 2. Assay was performed in vitro on exponentially growing cells. The activity was evaluated by measuring the levels of surviving cell after incubation for 24 h with the test samples, using the MTT colorimetric assay [15,17]. This is the first step in our anticancer drug development program and is designed to identify those extracts with cytotoxic activity. The results of this primary screening are reported in Figure 1. F2 exhibited a rather moderate cytotoxicity. 50% inhibition of cell growth was obtained at concentrations of 1225 and 980 μg/ml respectively against human tumor cell lines A549 and MCF7. However within the series studied, F3 revealed a significant activity against A549, HCT15 and MCF7 cell lines at concentration related manner (25–500 μg/ml), the results are shown in Figure 2. 50% inhibition of cell growth was obtained at concentrations of 231, 212.5 and 72 μg/ml respectively against human tumor cell lines tested A549, HCT15 and MCF7 (Table 2). Since the inhibitory effects of F2 and F3 on the inhibition of cell growth using the MTT colorimetric assay were established, we then examined their effects on cell viability by clonogenic inhibition assay against the same three human tumor cell lines (A549, HCT15 and MCF7) with the same range of concentrations. The latter allows the chemosensitivity study but is more time consuming than the other [18]. Results of this assay are presented in Table 3. In our experiments, data for antiproliferative effect of F2 on A549 and MCF7 cells showed a significant clonogenic inhibition at concentration-related manner, results are reported in Figure 3. 50% inhibition of cell growth was obtained at concentrations of 875 and 375 μg/ml respectively against human tumor cell lines tested A549 and MCF7 (Table 3). In a further experiment, F3 produced significant clonogenic inhibition too (Figure 4). IC_50_ are 137.5, 139.25 and 37.5 μg/ml respectively against human tumor cell lines tested A549, HCT15 and MCF7 (Table 3).

**Table 2:** **In vitro growth inhibitory activity of the semi-purified fractions, F2 and F3, of*Spongia officinalis:*against the three human tumor cell lines A549 (lung cell carcinoma), HCT15 (colon cell carcinoma) and MCF7 (breast adenocarcinoma)**

	**IC**_**50**_** (μg/ml)**		
Fractions	A549	HCT15	MCF7
F2 fraction	1225	-	980
F3 fraction	231	212.5	72

IC_50_: 50 percent inhibition of cell growth.

**Table 3:** **In vitro colony inhibitory activity of the semi-purified fractions, F2 and F3, of*Spongia officinalis*against three human tumor cell lines: A549 (lung cell carcinoma), HCT15 (colon cell carcinoma) and MCF7 (breast adenocarcinoma)**

	**IC**_**50**_**(μg/ml)**		
Fractions	A549	HCT15	MCF7
F2 fraction	875	-	375
F3 fraction	137.5	139.25	37.5

IC_50_: 50 percent inhibition of colony formation

Both F2 and F3 semi-purified fractions of *Spongia officinalis* showed, in vitro, significant antiproliferative activities. The IC_50_ values clearly indicated that the semi-purified fraction F3 had a much more potent effect, on the three human tumor cell lines tested, than F2. In term of cell line sensitivity, different responses were observed against the three human tumor cell lines, and no effect was observed on HCT15 cells with the semi purified F2.

## Discussion

Carrageenan rat paw edema assay is one of the most commonly used assays to assess anti-inflammatory activity of marine natural products from sponges [10,19]. Carrageenan rat paw edema test produced an acute inflammation that results from the sequential action of several mediators. Histamine and serotonin were mainly released during first 1.5 h after carrageenan injection, kinin was released until 2.5 h and at the last step inflammation was continued until 5 h by prostaglandins [20-22]. The anti-inflammatory activity of the methanol extract and its semi-purified fractions suggest that they could interfere with some of the mediators, by inhibiting their productions or antagonize their actions. Anti-inflammatory drugs such as aspirin and other NSAIDs act by downregulating prostanoids synthesis. Prostanoids are potent biologically active arachidonic acid derived lipid mediators that are intimately involved in inflammation and cancer [23]. In addition to their anti-inflammatory activity, *Spongia* have also cytotoxic properties. Related agents, such as macrolide [24], furanoditerpene [25], polyketide [26], alkaloid, sesterterpene, triterpene, furanoterpene [10,27,28], diterpene [29], sesquiterpene and nucleoside [30], have already been isolated from other species of the genus *Spongia*.

The functional tests to predict the response of tumors to cytotoxic drugs comprise the MTT reduction and clonogenic assay. The MTT colorimetric assay [15,17] is based on the ability of metabolically active cells to convert the pale yellow MTT to a blue formazan product, which is quantifiable spectrophotometrically and the clonogenic assay or colony formation assay is an in vitro cell survival assay based on the ability of a single cell to grow into a colony. The colony is defined to consist of at least 50 cells. This clonogenic assay has been used in the ensuing decades for a large variety of studies with many types of cells. The assay detect all cells that have retained the capacity for producing a large number of progeny after treatments that can cause cell reproductive death as a result of damage to chromosomes, apoptosis [31].

In the current study, both F2 and F3 fractions of the Mediterranean sponge, *Spongia officinalis* showed, in vitro, a significant antiproliferative activity against three human cancer cell lines A549, HCT15 and MCF7. The IC_50_ values clearly indicated that the semi-purified fraction F3 had a much more potent effect on the three human tumor cell lines than F2 and should be tested on several other cancer cell lines.

All these findings support the need for further investigations to clarify the features underlying the anti-inflammatory and the antiproliferative potential of these fractions. Biochemical and molecular studies carried out using the fraction in different animal models to establish their therapeutic efficacy, and subjected to HPLC and LC&MS analyses to identify and characterize the efficacious bioactive compound(s) in *Spongia officinalis*.

The authors acknowledge the “Ministry of Higher Education, Scientific Research and Technology, Tunisia”.

## Competing interests

The authors declare that they have no competing interests.

## Authors’ contributions

AD: made contribution to the study anti-inflammatory activities. MD: Was responsible for the conception and design, testing and data acquisition, analysis and data interpretation and drafted the manuscript. Audrey C-L: made contribution to anti-inflammatory activity. HBM made contribution to preparation of crude extract and its fractions of the defensive secretion from the Mediterranean sponge, *Spongiaofficinalis* and to their antiproliferative activities. AB made contribution to statistical analysis. All authors read and approved the final manuscript.
